# Origins of altered reinforcement effects in ADHD

**DOI:** 10.1186/1744-9081-5-7

**Published:** 2009-02-18

**Authors:** Espen Borgå Johansen, Peter R Killeen, Vivienne A Russell, Gail Tripp, Jeff R Wickens, Rosemary Tannock, Jonathan Williams, Terje Sagvolden

**Affiliations:** 1 Centre for Advanced Study (CAS) at the Norwegian Academy for Science and Letters, Oslo, Norway; 2 Department of Physiology, Institute of Basic Medical Sciences, University of Oslo, Oslo, Norway; 3 Department of Psychology, Arizona State University, Tempe, AZ 85287-1104, USA; 4 Department of Human Biology, Faculty of Health Sciences, University of Cape Town, Observatory 7925, South Africa; 5 Human Developmental Neurobiology Unit, Okinawa Institute of Science and Technology, Okinawa, Japan; 6 Neurobiology Research Unit, Okinawa Institute of Science and Technology, Okinawa, Japan; 7 Research Institute of The Hospital for Sick Children, University of Toronto, Canada

## Abstract

Attention-deficit/hyperactivity disorder (ADHD), characterized by hyperactivity, impulsiveness and deficient sustained attention, is one of the most common and persistent behavioral disorders of childhood. ADHD is associated with catecholamine dysfunction. The catecholamines are important for response selection and memory formation, and dopamine in particular is important for reinforcement of successful behavior. The convergence of dopaminergic mesolimbic and glutamatergic corticostriatal synapses upon individual neostriatal neurons provides a favorable substrate for a three-factor synaptic modification rule underlying acquisition of associations between stimuli in a particular context, responses, and reinforcers. The change in associative strength as a function of delay between key stimuli or responses, and reinforcement, is known as the *delay of reinforcement gradient*. The gradient is altered by vicissitudes of attention, intrusions of irrelevant events, lapses of memory, and fluctuations in dopamine function. Theoretical and experimental analyses of these moderating factors will help to determine just how reinforcement processes are altered in ADHD. Such analyses can only help to improve treatment strategies for ADHD.

## Background

Attention-deficit/hyperactivity disorder (ADHD) is one of the most common and persistent behavioral disorders of childhood, consisting of developmentally inappropriate, persistent, and impairing levels of hyperactivity, impulsiveness and inattention [1]. The prevalence of the disorder is similar in different cultures [2-4], about 5% of school-aged children [5] and 4% of adults [6] are affected worldwide. The disorder places the child at increased risk of school failure, juvenile delinquency, criminality, substance abuse, and HIV/AIDS as a consequence of sexual promiscuity and disregard for preventative measures [7-9]. For these reasons, the disorder is extremely costly to the afflicted individuals, their families, and to their society [10,11]. Despite being one of the most intensively studied psychiatric disorders, its etiology, diagnosis, and optimal treatment strategies are still the subject of debate and controversy.

Genetic factors have been identified [12], probably producing alterations in catecholaminergic regulation of brain function in frontosubcortical pathways [13,14]. At a behavioral level, children with ADHD respond atypically to reinforcers whether they are tangible rewards or social praise; they are less able to delay gratification and often fail to respond to discipline [15-21]. Compared to typically-developing peers, they perform less well under schedules of partial reinforcement [22,23]. Children with ADHD also respond more impulsively during delayed reinforcement in that they are more likely than typically-developing peers to choose small immediate reinforcers over larger delayed reinforcers [16,24-27]. Atypical response to reinforcement is a pervasive and fundamental characteristic of ADHD, which has important implications both for understanding the brain mechanisms underlying the disorder and for the development of effective behavioral and pharmacological interventions.

There have been many attempts to explain the origins of ADHD symptoms. A dual-process theory [17,28-31] suggests that less efficient reinforcement processes may explain several of the characteristic behavioral patterns. The temporal relationship between stimulus, response, and reinforcer strongly influences the effectiveness of reinforcers. For reinforcement to alter behavior, events need to occur within a limited time frame, but the extent of this time frame also depends on attentional and memorial variables. This is important both in basic laboratory research, where it is often overlooked, and in analysis of ADHD, which is associated with poor attention and memory [32,33].

This paper will first briefly describe the role of the catecholamines in response selection and memory formation before reviewing the neurobiological bases of reinforcement in general and discriminated response learning (i.e. stimulus-response-reinforcement learning) in particular. We then explore the delay-of-reinforcement gradient which describes the temporal window for the association of predictive cues with behavior and its consequences. Alterations in the shape of the delay gradient may be directly linked to dopamine dysfunction, but may also be secondary to changes in attention and memory processes. Further, we briefly describe how the core symptoms of ADHD can be explained by a steepened delay-of-reinforcement gradient. Finally, based on operant theory and empirical findings, we describe behavioral procedures for minimizing effects of a steepened gradient, and discuss challenges for reinforcement theories of ADHD.

## The role of catecholamines in response selection and memory formation

Behavior is guided by neural representations of previous experience. These memories are encoded in neural networks that represent the different elements of perception, motor response, and their consequences as well as the associated cues that predict the outcome. Increased synaptic efficacy, long-term potentiation (LTP), is commonly regarded as a prime candidate for mediating learning and memory [34,35]. Hebb proposed that connections between two neurons are strengthened when one neuron repeatedly or persistently takes part in firing the other neuron (presynaptic and postsynaptic activity being the two factors) [36], a proposition now known as *Hebb's rule*. The catecholamines dopamine and norepinephrine are required for selection and strengthening of responses that produce the reinforcer (reward). They also play an essential role in working memory (immediate, which lasts for seconds), short-term (lasts seconds to minutes) and long-term memory (lasts hours to years).

### Norepinephrine

The noradrenergic system is part of a coordinated structure that promotes behavioral adaptation to novel environments [37]. Noradrenergic neurons fire phasically in response to novel stimuli as well as to changes in environmental contingencies [37,38]. The norepinephrine projection to the prefrontal cortex is engaged by novel action-outcome contingencies, compatible with a role in mechanisms of plasticity and new learning [39]. At a cellular level, norepinephrine strengthens synaptic connections in neural circuits and thereby reinforces attention and memory [40].

### Dopamine

Dopamine is essential for both LTP and long-term depression (LTD) in brain areas that are critically involved in learning [41]. Dopamine activation of D1 receptors mediates reinforcement of behavior by strengthening synaptic connections between neurons (LTP) or weakening synaptic connections (LTD) in neural circuits that involve the prefrontal cortex and/or striatum (cortico-cortical and/or cortico-striato-thalamo-cortical circuits) [42]. Dopamine modulation of LTP is probably the neurobiological basis of reinforcement of behavior, whereas dopamine-induced LTD may be the mechanism that underlies extinction processes [17,43].

Response selection is sensitive to contextual factors. Input from the hippocampus gates the prefrontal cortex input and facilitates behavioral output based on the current context of the situation (time and place) or past experiences with the stimulus [42]. Dopamine projections to prefrontal cortex, hippocampus, and amygdala directly influence transmission in neural networks that involve these structures. Dopamine is further involved in memory processes by modulating neurons in the prefrontal cortex that are active during the delay interval between a stimulus presentation and a response [44], and may regulate working memory and attention [13,45].

### Neurobiology of reinforcement

Most investigators agree that mesolimbic and mesostriatal dopamine systems contribute to the psychological functions of reward – (incentive) and reinforcement-related learning, strengthening or increasing the probability of future occurrences of the behavior that preceded the reinforcer [46]. However, the exact role of these dopamine systems has been controversial.

Several associative processes occur during learning on the basis of positive reinforcement. These include classical conditioning (stimulus-stimulus association), habit formation (stimulus-action association), and learning of action-outcome contingencies. These processes are associated with activity in specific brain regions and can be shown to be selectively impaired by damage to those regions [47]. Here, we focus on discriminated response learning (learning that a response may be followed by a reinforcer only in the presence of a particular stimulus) which involves all of these processes.

A considerable body of evidence from single neuron recordings in monkeys indicates that dopamine cells fire phasically in response to unpredicted primary and secondary reinforcers [48,49]. Dopamine release brought about by phasic activity of dopamine neurons appears to be necessary for learning on the basis of positive reinforcement [50,51]. In particular, the majority of dopamine neurons show phasic activation after unpredicted primary reinforcers and conditioned, reinforcer-predicting stimuli (conditioned reinforcers), but not to aversive events which inhibit dopamine cell firing [48,52]. In these experiments, bursts of action potentials occur initially in response to a liquid reinforcer, then to solenoid clicks that precede delivery of the reinforcer. These clicks and other sensory cues associated with the primary reinforcer become secondary reinforcers. After training, the dopamine neurons fire at the occurrence of the earliest cue that predicts the reinforcer [48].

Rodent, primate, and human studies provide evidence that the striatum plays a key role in learning based on positive reinforcement [53]. In rats, lesions of the dorsal striatum impair acquisition of tasks requiring discriminated response learning. Behavioral measures in humans with neurodegenerative diseases of the striatum also provide evidence of its role in discriminated response learning [47].

Anatomically, the neostriatum is in a unique position to integrate the three factors of stimulus, response and reinforcement. The striatum receives input from nearly all areas of the cerebral neocortex in a topographical fashion [54]. The inputs from the neocortex make direct synaptic contact with the spiny neurons of the neostriatum. These neurons, in turn, project back to the neocortex via the thalamus. Hence, the nature of the corticostriatal inputs and the input-output relationship with the spiny projection neurons are the crucial determinants of striatal output. The neostriatum also receives input from the dopaminergic neurons. As noted above, the dopamine-producing neurons of the pars compacta of the substantia nigra display short periods of increased activity after the unpredicted presentation of food or liquid reinforcers and are believed to be involved in acquisition of behavior based on reinforcement. Nigral dopaminergic neurons project predominantly to the neostriatum where they converge with the inputs from the neocortex, amygdala and hippocampus. The convergence of dopaminergic and corticostriatal synapses upon individual neostriatal neurons provides a favorable substrate for a three-factor synaptic modification rule because it brings together the processes of three groups of cells (neocortical, neostriatal, and dopaminergic neurons). The three-factor synaptic modification rule was proposed as a cellular reinforcement mechanism for discriminated response learning, in which the situation is represented by the neocortical state (presynaptic activity), responses are represented by neostriatal neural activity (postsynaptic activity), and dopaminergic neurons encode reinforcing events (the third factor). The conjunction of these three factors was proposed to underlie learning by strengthening the synapses connecting the cerebral cortex to the striatum [55-57]. A conjunction of neocortical and striatal activity in the absence of the reinforcing dopamine signal was proposed to underlie extinction by weakening the active synapses [43].

Demonstration of the operation of a three-factor rule for synaptic modification in the dorsal part of the neostriatum was first reported on the basis of experiments in brain slices [58]. The three-factor hypothesis was tested by ejecting small pulses of dopamine to coincide with the conjunction of neocortical and neostriatal activity. Pulsatile ejection of dopamine, mimicking the effects of phasic reinforcer-related firing of dopamine cells, caused LTP of neocortical inputs. In the absence of pulsatile dopamine, LTD was induced. Thus, pulsatile dopamine stimulation activated a molecular switch that converted LTD into LTP [58]. More recently, it has been shown that the timing of back-propagating postsynaptic action potentials relative to arriving corticostriatal excitatory inputs determines whether LTP or LTD takes place and that dopamine receptor activation is required for both LTD and LTP induction [59].

The functional significance of this three-factor rule is that striatal projection neurons effectively encode the integrated history of reinforcement of actions performed in specific situations. The effectiveness of their synaptic inputs from the neocortex translates the current cortical activity pattern into a value representing the probability of reinforcement. This is because each instance of reinforcement produces an incremental increase in the effectiveness of the contributing synapses, so their effectiveness comes to represent the integrated history of reinforcement over time. Since these are excitatory inputs, their effectiveness is translated into depolarization of the postsynaptic neurons, the activity of which provides a readout of the expectation of reinforcement in the particular context of cortical activity [60,61].

The three-factor rule, in the context of the corticostriatal pathway, provides a plausible cellular mechanism for selecting responses that have been reinforced in the past. The three-factor rule operates within a limited temporal window. Reinforcer-related release of dopamine must coincide with synaptic activity representing behavior and the situation within a short (subsecond) time interval for LTP to take place [62]. In the following section, we examine the temporal constraints of the three-factor rule from a behavioral perspective. The delay-of-reinforcement gradient is a central concept in operant theory, elaborating the three-factor rule along a temporal dimension. In the following section, the origin of the delay-of-reinforcement gradient, its direction, and its relation to attention and memory processes are discussed. Knowledge of the various components and processes feeding into the reinforcement and response selection processes is important when investigating how reinforcers act.

## Delay of reinforcement – decay gradients

A reinforcer is not defined in terms of previous events; it is defined in terms of what happens next – by the behavioral changes that follow reinforcement. Reinforcers act on responses in the same class as those that preceded their presentation, within a limited time frame (seconds) from the occurrence of the behavior to the perception of its consequences. This is the case for humans along with other animals; but for humans that brief window may be enormously expanded by verbally formulated prompts or rules, such as *Last time when I did my homework, I got a smile from the teacher; I want that again*. In turn, the utility of such prompts depends on the ability of the individual to keep them active in mind and use them to guide behavior. Delays between action and outcome impair conditioning [63]: The strength of a discriminated response association is inversely related to the delay between the response and the reinforcer [64-66]. This does not mean that strength dissipates over delay; reinforcers strengthen the response state that the animal is in; effects of delay arise because the longer the time between the response and the reinforcer, the more likely it is that the animal has left the behavioral state it was in when it responded, so some other state, i.e. some other neuronal activity, will be erroneously reinforced [67]. The weakening of conditioning with delay is a credit allocation problem; precise allocation becomes more difficult with delay.

### The origin of the gradient

Reinforcement strengthens association between a stimulus and response, context and consequence. When in the context, presentation of a discriminative stimulus prepares the organism for reinforcement, and may cause the rate of responding to increase, consistent with Skinner's definition of reinforcement [68]. The process may also occur through classical conditioning (Pavlovian S-S association), causing the context, or predictive stimuli within it, to become associated with reinforcement, and thus to become attractive to the organism. There is a problem however: A stimulus or response which occurs some time before a reinforcer cannot contemporaneously be joined with the reinforcer. The stimulus or response must leave some kind of trace that is present at the time of reinforcement. This trace may be conceptualized as a memory, a representation, a synaptic flow, or a reverberating circuit. How are such representations of appropriate stimuli and responses formed? The prior section proposes some neurobiological mechanisms; here we consider parallel behavioral mechanisms.

**Figure 1:**
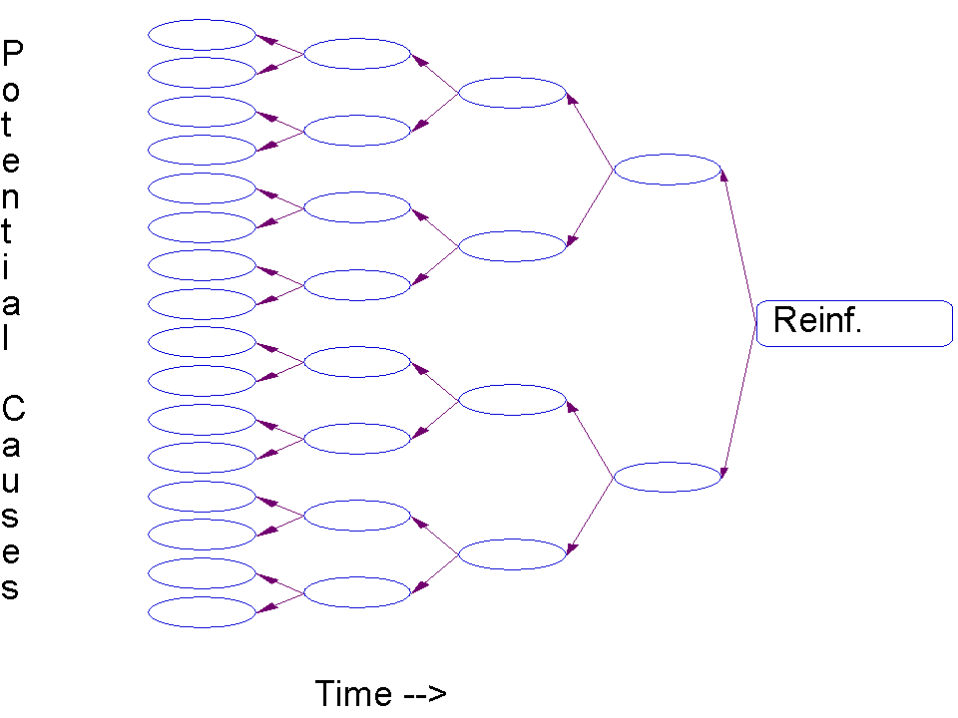
**Reinforcers do not act backwards in time, but on memory representations of preceding causes**. Candidate causes proliferate as an effect is delayed. Absent special clues, this leads to a geometric decrease in credit that is available to be allocated to any one of the precedents.

Consider the string of events experienced by the organism, leading up the current position in time (Figure 1). Several events occur and then a signal event – a reinforcer – occurs. How is credit for the reinforcer allocated to precedent events? It is clear that immediately before the reinforcer there are only one or a few candidate events, and that their number grows exponentially as we back away. Not only must the multiple candidates be evaluated, but the complete path between them and the reinforcer must be considered. Even in an impoverished world with only two states, as shown in Figure 1, the number of candidate histories grows as 2^*n*^, where *n *is the number of steps back in time that we consider. It follows that the attention that the organism pays to each path – the credit assigned to each – must decay geometrically with the time to the reinforcer.

**Figure 2:**
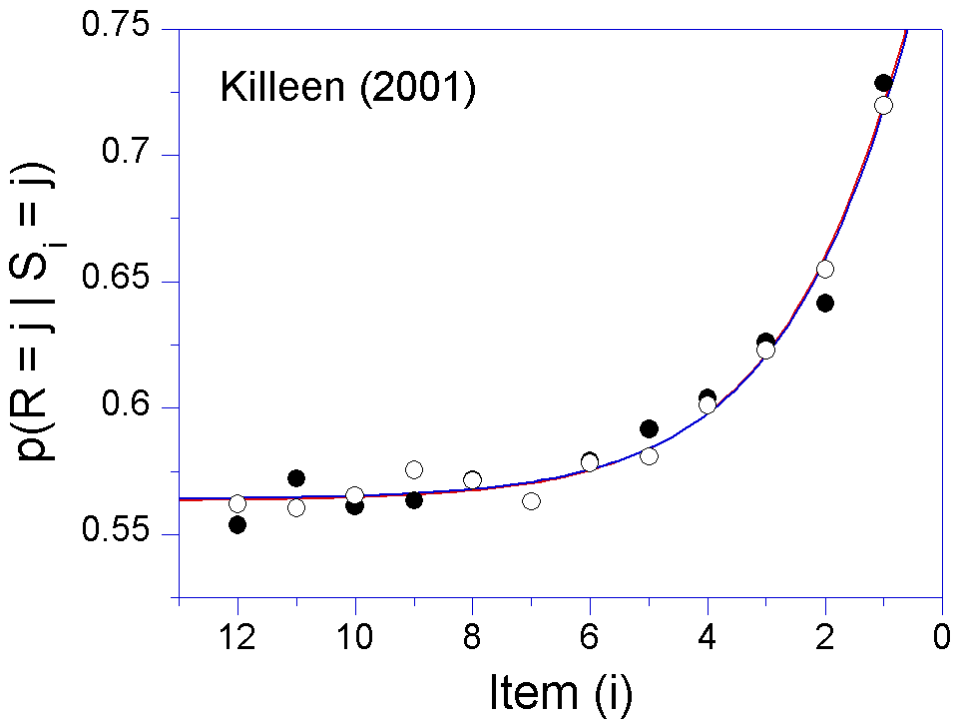
**The decay in the influence of a stimulus as a function of the number of items intervening between it and reinforcement **[69]. The ordinates are the probability that a summary response that indicated the preponderant color (e.g., "mostly red") was the same as the color presented in the ith position. Each element was presented for 425 ms. Filled circles: The time between stimuli (ISI) = 75 ms; Open circles: ISI = 425 ms. The memorial part of the model was a geometric decay, with rate = 0.4 per item. In this figure a comparable exponential function (Equation 1 in Appendix 1) was used to generate continuous forgetting functions. The data are averages over 5 pigeons.

Consider the rudimentary case where a series of red and green lights are flashed, too quickly to count, and the subject is required to judge whether the sequence was predominantly red or green [69]. Here the delay between each element is subject to the decay equation (see Appendix 1), because interposed between it and the end of the trial (and possible reinforcement) lies a series of other flashes which cannot be ignored. We may measure the importance of each element in the sequence by plotting how often the response (judgment) corresponded to the color of that element. Of course, sometimes the judgment was correct, sometimes incorrect, depending on the context of other colors. But averaged over these randomized presentations, it tells us how much credit was given to each element (Figure 2).

The influence of events decreased rapidly with each interposition, approaching asymptote after 6 items. Note that an almost six-fold increase in the time between stimuli had no affect on the rate of memory decay: Events, not time, caused most of the forgetting in this study. Similar results were reported by Waugh and Norman [70]. *It is obvious that time is often not the proper independent variable in the study of memory decay; it is the number of events processed per unit of time that matters*. Since in the flow of the real world the number of events is often inscrutable, however, time is often taken as its proxy. Outside the laboratory, experience and time are intrinsically correlated, so the common assumption that memory, and its neural substrates, decay over time, is true. It is also pragmatic, because, unless they are carefully manipulated by an experimenter, the stimuli and responses that fill the delay interval are more difficult to measure than the interval's temporal extent.*But it is events in time, not time per se, that function as causes*. The steeper delay gradients that often characterize hyperactive organisms may be due to the greater number of events they expose themselves to during delays.

### The direction of the delay-of-reinforcement gradient

We may plot the decrease in associative strength at temporal removes from reinforcement as in Figure 2. This is the classic delay of reinforcement gradient. It is misleading, however, if construed as mechanism. It is the ability to hold the response (and the associated stimuli) in memory that decays over time; reinforcers act on the decreasing tails of these memorial traces. Because those gradients decrease with time, they start at a near-maximal value at the time of the stimulus or response, and decay until the moment of reinforcement (the mirror image of the traditional representation). Gradients such as those shown in Figure 2 are a summary report of these processes, not the processes themselves.

The classic view is useful in the case of establishing new behavior with delayed reinforcers [17,29,71]; but it can be misleading when applied to the delay-of-reinforcement experiments so often utilized in the study of impulsivity. The underlying hypothetical gradients are often viewed as the strength of the pull toward the large-late or small-soon reinforcer, with the choice of the latter called impulsive, and explained in terms of steeper discount gradients [72,73]. If you judge a bird in the hand to be worth two in the bush, you are prudent; but if you think it worth four in the bush, you are impulsive. But the temporal discounting involved in such choice may have little to do with the steepness of trace gradients. In experiments with humans, the outcomes are presented verbally [64,74,75], and the obtained preferences and discount gradients are strongly influenced by the individual's ability to imagine these future situations, and relate them to his current desires. It is not so much a future event that is discounted, as the future self who will enjoy it.

### Delay gradients, working memory, and attention

As Henri Bergson [76] noted, perception and consciousness did not evolve to provide entertainment, but to prepare us for action; that action is shaped by reinforcement. Reinforcement cannot act backward in time, but only on the palette of events carried to it by memory. Each new event crowds in to overshadow the traces of older memories. It overwrites them, to be overwritten in turn, and again with each new step through time and the events that time carries. Rich environments present the potential for a disastrously quick loss of ability to allocate credit to the correct precedent. For how long do you typically retain the name of a newly introduced person, when this is accompanied by their novel appearance and personal details, in a general context of other novelties?

Memory is a key player in these analyses, and the variety of memory that is most relevant is working memory. Working memory capacity characterizes the ability to hold and utilize elements of information in a computation or action after they are briefly presented, with key elements under threat of displacement by the manipulation of them or by other events. Think of doing multiplication problems in one's head, or remembering a phone number while engaging in the ongoing conversation. This ability to hold, or retrieve, a representation, may underlie our ability to learn through reinforcement. Reduced memory capacity would functionally steepen the delay-of-reinforcement gradient, because fewer of the behaviors and events preceding the reinforcer are represented in memory at the time of reinforcement.

It may seem that simple conditioning should not require such representation. This may be the case for delay conditioning, where the stimulus overlaps with the reinforcer [77]. Awareness, however, is necessary for trace conditioning, where the stimulus is episodic and must be remembered during the delay [78,79]. Trace conditioning engages additional areas of the neocortex than the simpler delay conditioning, in particular areas that maintain working memory processes [80].

Attention is related both to reinforcement and to working memory. The strength of the memory trace at the time of reinforcement depends on the attention originally allocated, the number of competing states, and the relative salience of each of the ensemble of traces. In an unfamiliar situation, attention is captured by novel, salient stimuli in a bottom-up, memory-free way (yesterday's *orienting response*, today's *automatic capture of attention*) [81-83]. As the process of reinforcement unfolds, predictive stimuli or responses are recognized and become established as discriminative stimuli: Relevant behaviors will be performed in the presence of the discriminative stimulus, while other behaviors will be avoided. In these situations, specific stimulus properties are actively attended to because they signal favorable consequences [82,83]. When a stimulus has acquired discriminative properties, attention is guided in a controlled, memory-dependent way, as a learned behavior shaped and maintained by reinforcement [82,83]. The consequences of attending thus change what is attended to [84,85]. A familiar example is the Wisconsin card sorting test where positive consequences are arranged for attending to one of the three dimensions on the stimulus-cards (number, shape, or color) [86]. The consequences change which dimension the testee attends to and sorts by. Rats attend to the light signaling which of two response alternatives will produce a reinforcer; people attend to the wheels on a slot-machine because they signal when money is won; researchers attend to which of the granting agencies is in political favor because that shapes the flavor of the application.

## Reinforcement processes in ADHD

Forty years ago Wender suggested that reinforcers work differently in ADHD [19]; a fact known implicitly by parents long before that landmark book. Numerous studies have investigated effects of reinforcers in ADHD, and although the findings are not entirely consistent, reinforcers seem to affect behavior differently in ADHD than in control subjects (see [87] for a review). Rapid advances in neurobiology and genetics have produced compelling evidence for deficits in catecholamine functions in ADHD [13,14,17]. These findings, combined with research showing the importance of the catecholamines in memory and response selection processes [39,40,45,49-51], and especially of dopamine in behavioral acquisition based on reinforcement [88], support the early suggestion of a reinforcement deficit in ADHD [18-23,26,28,29,71].

Reinforcement and extinction processes are the fundamental mechanisms of behavioral selection [68]. This process is in many ways similar to selection in genetics: "Within the lifetime of the individual organism, behavior is selected by its consequences, much as organisms are selected over generations by evolutionary contingencies" [89]. To survive, organisms must generate novel behavior with yet unforeseen consequences and be able to profit from experience by increasing the frequency of successful responses and eliminating unsuccessful or maladaptive behavior. Reinforcement will strengthen preceding behavior regardless of whether the behavior is correct or incorrect [90]. A reinforcer presented after four incorrect responses followed by a correct response will strengthen both the incorrect responses as well as the correct response. However, because reinforcers are presented contingent on successful (correct) and not on unsuccessful (incorrect) responses, only correct responses will consistently precede reinforcers. Hence, in the long run, correct responses will be strengthened substantially more than the other responses. Mechanisms of behavioral selection must be sensitive to contextual factors; adaptive behavior in one context may not be adaptive in another. Habits, skills, and beliefs are sedulously built from simple behavioral units to longer behavioral sequences that come under the control of environmental stimuli (stimulus control) as reinforcers are delivered in some situations and not in others [17,91-93].

Human behavior is sometimes controlled, not by reinforcement contingencies, but by verbally formulated rules about the reinforcement contingencies and what the person believes is the correct/incorrect behavior. In these cases, the rules (Bacon's *Idols of the Marketplace*) may prevent behavior from making contact with the real contingencies of reinforcement.

### A steepened delay gradient in ADHD – symptoms

The symptoms observed in ADHD have been explained as an executive dysfunction [33,94,95], as a non-optimal mental energy state [96,97], as delay aversion linked to motivational deficits, and as a cognitive inhibitory deficit [26,95,98]. Above, we have described how the three-factor rule of reinforcement relevant at the cellular level can be translated into the delay-of-reinforcement gradient operating at a behavioral level. The delay-of-reinforcement gradient provides a way to describe how reinforcement processes are altered in ADHD. Changes in these behavioral selection mechanisms will inevitably produce behavioral changes. A steepened delay-of-reinforcement gradient can make sense of many of the behavioral symptoms associated with ADHD.

Several hypotheses and theories have been proposed on how reinforcement processes are altered in ADHD relative to normally developing children [18-23,26,28,29,31,71]. The dynamic developmental theory of ADHD posits that dopamine hypofunction in ADHD narrows the time window for associating predictive stimuli with behavior and its consequences [17,29]. This narrowed time window entails a steepened delay-of-reinforcement gradient. However, as previously described, it is events in time and not time itself that drives the delay-of-reinforcement gradient. Also, as shown in previous sections, both attention deficits and more rapid memory decay may cause steepening of the delay-of-reinforcement gradient in ADHD [99]. These perspectives represent an extension of the dynamic developmental theory.

Due to the association between dopamine and LTD, the theory also proposes that extinction processes are depressed in ADHD, causing a slowed or deficient elimination of previously reinforced behavior [17]. Altered reinforcement learning described by a steepened delay-of-reinforcement gradient combined with deficient extinction can produce the main symptoms of ADHD: Inattention, hyperactivity, impulsivity, and additionally increased behavioral variability [17,29,92,93,100-102]. Slowed learning of discriminative stimuli due to the steepened delay-of-reinforcement gradient leads to a weaker control of behavior by contextual cues: Behavior is not controlled over extended periods of time by the discriminative stimulus and may be inappropriate for the current situation [103]. This may be observed as symptoms of deficient sustained inattention (e.g. forgetful in daily activities; easily distracted; fails to finish schoolwork, chores, or duties in the workplace) [17].

Reinforcers also strengthen the temporal relation between consecutive responses or behavioral elements. A steepened delay-of-reinforcement gradient implies that mainly fast response sequences are reinforced. Hence, hyperactivity is suggested to be caused by the reinforcement of bursts of correct responses combined with deficient extinction of non-functional or incorrect behavior. Further, a steepened delay-of-reinforcement gradient signifies that delayed consequences of behavior have less effect in children with ADHD than in normal controls. Thus, poorer control of behavior and less effective learning would be expected with delayed reinforcement compared to that seen in individuals without ADHD. This prediction is consistent with the preference for immediate reinforcers reported in children with ADHD compared to normal controls [16,24-26,104].

The dynamic developmental theory of ADHD suggests that changes in fundamental behavioral selection mechanisms slow the association ("chunking") of simple response units into longer, more elaborate chains of adaptive behavioral elements that can function as higher-order response units [17,92,93,102]. When response units are chunked together into a chain, one response unit reliably precedes the next and there is a high degree of predictability within the response chain. Deficient or slowed chunking of behavior means that the reliable and predictable pattern of responses is absent, resulting in the increased intra-individual variability observed in ADHD [92,93,105,106].

The operant principles used to explain ADHD behavior in terms of a steepened delay-of-reinforcement gradient offers some suggestions on how to optimize learning in individuals with ADHD. These general suggestions are based on operant theory and empirical findings from studies of animals as well as humans. However, while these suggestions may be highly relevant for clinical interventions in ADHD, they are not specific, nor necessary all tested, in ADHD.

### Optimizing learning

A steepened delay-of-reinforcement gradient hampers learning and may lie at the core of the behavioral changes seen in ADHD. Interventions aimed at making the delay-of-reinforcement gradient functionally shallower will improve learning and reduce ADHD symptoms. The gradients become functionally shallower – have greater ability to capture more remote events – if: (1) there is minimal post-event interference; (2) the event persists – stimuli bridging the delay in the case of stimulus events, repetitive responses in the case of response events – so that later or similar parts of the event are close to reinforcement; (3) the event is marked for special attention; and (4) it precedes other events which have themselves become conditioned reinforcers.

#### 1. Interference

Post-event interference can be minimized by provision of a minimally disruptive context, or by the subject's ability to focus on a relevant subset of the environment. Retroactive interference is equally disruptive in human and non-human animals [107]. It is demonstrated in Figure 2 by the similarity of the two forgetting functions on an event axis; on a real-time axis the condition with the brief time between stimuli (inter-stimulus interval, ISI) would appear to decay at about twice the rate of the long ISI condition. The difference is that nothing was happening during the longer ISIs to disrupt memory, and any decay there was occurring at a much slower rate. Individuals with deficits in ability to allocate attention, whether toward long-term goals or simply away from immediate temptations – will be especially subject to interference, and therefore evidence steeper delay-of-reinforcement gradients. A major clinical challenge is, of course, to increase the subject's focus on relevant stimuli and minimize the disruptive context. Enhancing the salience of stimuli by e.g. the use of colors (see below) may increase the focus on relevant environmental factors. Additionally, and consistent with established educational practice, breaking up tasks into small and manageable parts may reduce effects of disruptive context and lead to improved learning and performance.

#### 2. Creating robust memory traces

In *delay conditioning*, a stimulus is continuously signaled during the delay to reinforcement. In *trace conditioning*, a stimulus is only briefly present, then removed during the delay. The former is many times more effective over moderate and long delays than the latter [108]. Thus, from a practical perspective, providing cues or stimuli that are continuously present during the delay to reinforcement may reduce demand on memory and improve learning and performance. Some memorial tactics essentially turn trace conditioning into delay conditioning, thus bridging the temporal gap. The extent to which individuals can do this constitutes their working memory capacity. Repetition of the event, either until it is needed, can be used, can be written down, or seeps into long-term memory, helps keep the memory of the stimulus alive for association. We repeat new phone numbers until a pen is found. Prey animals often keep the stimulus alive by paradoxical "stalking" of a predator, keeping it in sight so that memory of its presence will not be over-written by foraging. Neonates may bridge the gap with repeated stereotyped movements appropriate to the conditioned stimulus [109]. These tactics mark the trail through the labyrinth of Figure 1 from the initial event to the eventual reinforcer, just as seasoned hikers will periodically turn around to make a mental image of the trail they must choose to find their way back home.

#### 3. Attentional loading

Novel events catch our attention. Such attention can be enhanced by remarking on the novelty, as we might repeat the name of the new acquaintance when first introduced. A subsequent reinforcer then has the highlighted memory on which to work. This tactic winnows the candidate paths by weighting a particular event more heavily than contemporaneous stimuli. *Unpredicted events are noticed; unpredicted reinforcers capture attention and cause learning*. Predicted events and reinforcers are not further associated, and fade from attention. This gamble of vesting attention is often successful, but is never without risk, since distraction from relevant stimuli will hamper learning. Attention is the gatekeeper that decides which events enter memory to be candidates for reinforcement. Changes in attention-processes will affect the shape of the delay-of-reinforcement gradient. Thus, attention deficits in ADHD may be the primary culprit behind many of the other symptoms. However, attention is itself a behavior that is modifiable by reinforcement [84,85]. Thus, in the dynamic system that is a developing human being, the cause-effect status of attention versus reinforcement and learning is a chicken-and-egg problem; deficits in either will cause problems for the other, and interventions that help one will improve the other.

A tactic that is sometimes useful in determining the cause of a reinforcer post-hoc is to reduce the number of candidate events. We will often "concentrate": Become quiet and focused in our attention. Then we replicate as best we can a subset of potential candidates. The car seems to make a noise when we turn a corner. The next corner we turn, we do so with full attention: Does it replicate? Is it the steering gear or the mudguard? Does it happen in the other direction? This is at the heart of science: Minimize distraction and confounds, control and replicate, with careful documentation of the variables that were manipulated. Individuals with compromised attentional abilities may learn some of these skills as they mature, buffering the severity of those deficits. White [110] has convincingly argued that remembering is best considered as discrimination at the time of retrieval; events that are more likely to be reinforced support better discriminations.

**Figure 3:**
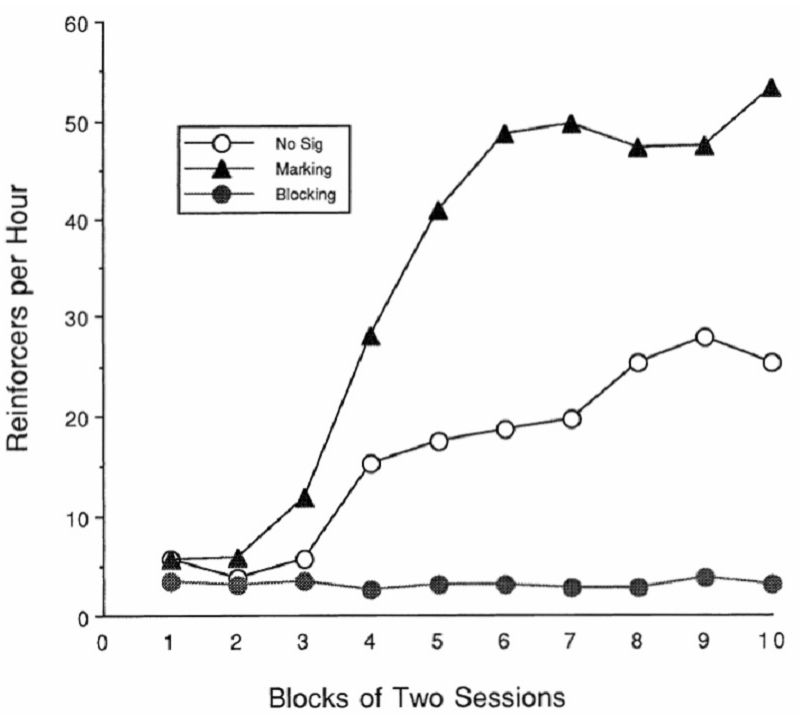
**Data from Williams **[113]**showing the efficiency of learning under two manipulations of attention and a control condition**. Reproduced with permission of the author and the Psychonomic Society.

Another memorial tactic is to bias the search for events that might typically produce the reinforcer. Hume [111] noted, and psychological research has validated [112], that similarity strengthens association, as does spatial proximity. Indeed, the fan-out in Figure 1 is really a function of space-time. The larger the spatial context that must be considered, the more events must be processed at each step. This is vividly demonstrated in an experiment by Williams [113], who trained rats to lever-press with a 30 s delay between the response and reinforcement. The Marking group (triangles in Figure 3) received a 5 s chamber illumination immediately after the lever-press; the Blocking group (filled circles) received a 5 s chamber illumination immediately before the reinforcement; the control group received no chamber illumination. Figure 3 shows the results of his experiment.

The results are remarkable in two ways. In the case of blocking, a prominent stimulus essentially absorbed all of the credit for reinforcement, leaving none to strengthen the originating response. A simple-minded application of the principle of conditioned reinforcement – "*Here's a nice reward for you Johnny*" might effectively undermine the strengthening of the very response it was intended to enhance! Reminding the individual of the relevant response at the time of reinforcement can restore some of that potency. In the case of marking, the results endorse the wisdom of the adage "Catch them being good". It is likely that much of the efficacy of what we call conditioned reinforcement is due, not to conditioned reinforcement, but to marking. Marking relevant stimuli is especially important for individuals with attentional deficits. Some protocols for helping children learn involve gesture, voice modulation and visual marking to increase salience of relevant information, and precueing the desired behavior at point of performance, which then permits immediate feedback – reinforcement – that can be integrated with the target behavior. Rowe [114] encourages the use of stimulus dimensions, such as color, that increase the salience of the discriminative stimuli.

#### 4. Backward chaining

A leading model of conditioning, the temporal-difference (TD) model [115], has proven successful in machine-learning instantiations, and has been seminal in the study of brain correlates of learning [116]. This model essentially vests a proportion of the reinforcing strength of the primary reinforcer in each of the states that precede it, one step at a time on each occasion of conditioning. Such backward chaining prunes the causal net of Figure 1. It is a classic approach to establishing long sequences of behavior [117]. Due to a steepened delay-of-reinforcement gradient, children with ADHD may have problems chaining responses into adaptive behavioral sequences where the elements in the sequence are linked together and function as a higher-order response unit (e.g. have difficulties finishing schoolwork, chores, or duties in the workplace without a long "to do list" or reminder notes). From an applied perspective when working with children, backward chaining and other behavioral techniques aimed at building or increasing sequences of behavior may be useful in ADHD and other developmental disorders in one-on-one settings. This strategy is often inconvenient to use for the rapid transmission of information in classroom settings. However, effective educational programs have been developed where sequences of behavior are built through the use of a strategy termed "scaffolding" where the teacher models, prompts, and reinforces behavior in a step-by-step fashion until the child performs the whole sequence independently, accurately, and fluently [118]. Scaffolding is a component in the Tools of the Mind curriculum which has been shown to enhance learning, executive functioning, and development in preschool children [119,120].

### Challenges for reinforcement theories of ADHD

Given the heterogeneity of ADHD findings, it is unlikely that any one theory can explain all cases of ADHD. Nevertheless, theories of ADHD should enable the integration of data from behavioral, genetic, neurobiological, cognitive, and clinical studies of ADHD. Reinforcement theories can explain many of the symptoms associated with ADHD and link these behavioral changes to changes at genetic and neurobiological levels through deficiencies in how the neuromodulator dopamine works. In this paper, we have also shown how cognitive processes like memory and attention are linked to the effects of reinforcers and may lie at the base of the suggested steepened delay-of-reinforcement gradient in ADHD. However, it is sometimes forgotten that also "top-down control of behavior" is acquired through learning. Cognitive processes like working memory, attention, and executive functions do not represent permanent traits of the individual, but are processes that can be significantly improved by training [84,85,121-125]. These findings attest to the importance of the environment in shaping and maintaining these functions. Hence, the primacy of these cognitive functions versus basic learning mechanisms and the directionality of cause and effects in ADHD need to be further studied.

A challenge for reinforcement theories of ADHD is to link the concepts of memory and attention used in our analyses of behavior to the corresponding concepts used in cognitive psychology. ADHD is associated with cognitive deficits including working memory impairment [32,33]. However, a precise translation from behavior to cognition requires a better operationalization of concepts such as short-term memory, long-term memory, working memory, encoding, storage, retrieval, attention than is currently available.

Previous studies of reinforcement processes in ADHD have used a variety of experimental designs and methods, producing a fragmented research literature. The reinforcement universe is broad and includes several important dimensions like reinforcer density, reinforcer delay, reinforcer predictability, and reinforcer value. The research questions become yet more challenging when the influences of memory and attention processes are taken into account, as they must be. Future studies need to systematically explore the various mechanisms that can affect the delay-of-reinforcement gradient, whether they are functionally equivalent and produce similar symptom outcomes, or whether they give rise to endophenotypes that can be differentiated and identified. Exploring possible common causative mechanisms, like deficient memory processing, may provide an opportunity for the integration of a reinforcement deficit as a causative factor with the complex network of other causal factors suggested for ADHD [126-133].

The section on optimizing learning by minimizing post-event interference and increasing attentional loading by marking of events suggests future studies of ADHD. The effects of time versus intervening events on memory decay and reinforcement effects in ADHD compared to normal controls can be tested using Killeen's procedure [69] modified for human subjects. Further, results from this procedure can be compared with data from studies using delayed-matching-to-sample data, i.e. time-driven memory decay (e.g. [134]), and studies of effects of interference in ADHD, i.e. event-driven memory decay (post-event interference, events occurring following the to-be-remembered event, should not be confused with interference tested by Stroop tests; the slowed response-time due to the suppression of an automated response, e.g. [135]). Additionally, reinforcer valence/magnitude can be varied to test whether this is independent of the obtained decay functions. The importance of attentional loading on memory and reinforcement effects can be tested by varying the salience of the stimulus used for response-marking, vary the temporal relation between the response and the marking stimulus, and possibly also test response marking combined with reinforcer delay to explore the memory decay of the marked response [136].

Reinforcement theories of ADHD need to explain not only the development of symptoms and the relation to other levels of description, but also the improvement of behavior following psychostimulant treatment. A challenge for such theories is that the symptom-reducing effects of central stimulants in ADHD seem too rapid in onset to be plausibly attributed to learning [137]. Further, if drugs improve learning, then behavioral improvement should be long-lasting. However, the major beneficial effects of the drug wear off within hours, and correction of a learning deficit per se may seem an unlikely mechanism for these drugs' therapeutic actions. However, any medication that alters a reinforcer's effectiveness will shift the relative likelihoods of different classes of behavior, potentially producing rapid behavioral changes [101]. In this sense, medication does not supply what the child has failed to learn in the past; it merely makes the child more able to attend and control his behavior under medication. This assumes that appropriate behavior is in the repertoire of children with ADHD, but is not produced due to the prevailing motivation or reinforcement contingencies. This is consistent with the observations that children with ADHD show adequate behavior under some reinforcement contingencies (continuous and immediate reinforcement) but not under other contingencies (partial and delayed reinforcement), and is consistent with the clinical notion that ADHD is not a problem of "knowing what to do but one of doing what you know" [94].

## Conclusion

The three-factor rule describes an important principle underlying discriminated response learning at a synaptic level. Synaptic strengthening depends on the convergence of dopaminergic synapses (representing reinforcers) and corticostriatal synapses (representing the stimulus situation) upon individual neostriatal neurons (representing behavior) [56,57]. The three-factor rule can be translated into the delay-of-reinforcement gradient which is a concept operating at a behavioral level. Alterations in reinforcement processes in ADHD may be described by a steepened delay-of-reinforcement gradient which can explain the development of symptoms of inattention, hyperactivity, and impulsivity associated with ADHD [17,29]. The shape of the delay-of-reinforcement gradient is influenced by several processes, in particular attention and memory. Theoretical and experimental analyses of these factors are important to determine if and how reinforcement processes are altered in ADHD. Such analyses could also promote the collaboration between research groups, facilitate an integration of the ADHD research field, and ultimately lead to improved treatment strategies and intervention programs for ADHD.

## Competing interests

The authors have no competing interests and are listed in approximate order of individual contribution to the manuscript.

## Authors' contributions

All authors contributed to discussions, helped to draft the manuscript, and read and approved the final version of the manuscript.

## Appendix 1

### The delay of reinforcement gradient as diffusion of attention

If 4 states must be considered for credit as a cause of a reinforcing or punishing event at each step of a sequence of prior events, for *n *steps there are 4^*n *^paths; for six states, 6^*n*^, and for *a *states, *a*^*n*^. If the total credit available is *c*, and if it is to be evenly distributed, then the credit allocated to each path is *ca*^-*n*^. For consistency with traditional continuous models, replace *a *with the base *e *= 2.718... and *n *with λ*t*, with *t *the time until reinforcement. The rate constant lambda (λ) is then the natural logarithm of the number of states evaluated per second (λ = ln[*a*]). If the total credit available is *c*, and if it is evenly distributed, then the credit allocated to each path must decay as:

(1) *s *= *cλe*^-λ*t*^

Note that **λ **re-emerges as a coefficient in Equation 1. That is because, under the assumption of constant capacity *c*, the area under Equation 1 is conserved, so that its integral must equal *c *independent of the rate of allocation of attention. Equation 1 satisfies that assumption; steepening of the gradient associated with increases in lambda will also increase its intercept (*cλ*). Other assumptions are possible. Suppose, for instance, that an initial credit *c *is depleted at a rate of *λ*, with no assumption of invariance of capacity over its rate of allocation. Then the appropriate model is:

(2) *s *= *ce*^-λ*t*^

In this case the function is "hinged" at an intercept of *c*. It is an empirical question which of these models is most relevant to research on ADHD [100]. Because capacity *c *is often a free parameter, the difference between the two models is blunted by the models' ability to absorb λ into *c*: *c' *= (*cλ*). The test will be to see whether, by varying the number of states or their rate of presentation, the resulting changes in *λ*, are correlated with changes in *c*. If Model 1 is correct, but Equation 2 is used, then there should be a positive correlation between *c *and *λ*.

If the exhaustion of credit is modeled by Equations 1 or 2, those equations, ceteris paribus, also tell us how strongly a remote event is likely to be associated with reinforcement. But they do not tell the whole story, because they leave out the factors of marking, similarity and context. Modifications of this model are straightforward, but await relevant data.
